# Establishment of in-house assay for screening of anti-SARS-CoV-2 protein inhibitors

**DOI:** 10.1186/s13568-024-01739-8

**Published:** 2024-09-16

**Authors:** Merna H. Emam, Mohamed I. Mahmoud, Nadia El-Guendy, Samah A. Loutfy

**Affiliations:** 1 Nanotechnology Research Center (NTRC), the British University in Egypt, Suez Desert Road, El-Shorouk City, P.O. Box 43, Cairo 11837 Egypt; 2 School of Biotechnology, Badr University in Cairo, Badr City, 11829 Cairo Egypt; 3 Medical biochemistry and Molecular biology unit, Cancer Biology Department, National Cancer Institute (NCI), Cairo University, Fom El-Khalig 11796, Cairo, Egypt; 4 Virology and Immunology Unit, Cancer Biology Department, National Cancer Institute (NCI), Cairo University, Fom El-Khalig 11796, Cairo, Egypt

**Keywords:** Protein-protein interaction, SARS-CoV-2, Spike, ACE2, Immunofluorescent immunoassay, Natural compounds

## Abstract

Developing a potent antiviral agent to combat Coronavirus Disease-19 (COVID-19) is of critical importance as we may be at risk of the emergence of new virus strains or another pandemic recurrence. The interaction between the SARS-CoV-2 spike protein and Angiotensin Converting Enzyme 2 (ACE2) is the main protein-protein interaction (PPI) implicated in the virus entry into the host cells. Spike-ACE2 PPI represents a major target for drug intervention. We have repurposed a previously described protein-protein interaction detection method to be utilized as a drug screening assay. The assay was standardized using Chitosan nanoparticles (CNPs) as the drug and SARS-CoV-2 spike-ACE2 interaction as the PPI model. The assay was then used to screen four natural bioactive compounds: Curcumin (Cur), Gallic acid (GA), Quercetin (Q), and Silymarin (Sil), and their cytotoxicity was evaluated in vitro. Production of the spike protein and the evaluation of its activity in comparison to a standard commercial protein was part of our work as well. Here we describe a novel simple immunofluorescent screening assay to identify potential SARS-CoV-2 inhibitors that could assess the inhibitory effect of any ligand against any PPI.

**Graphical Abstract:**

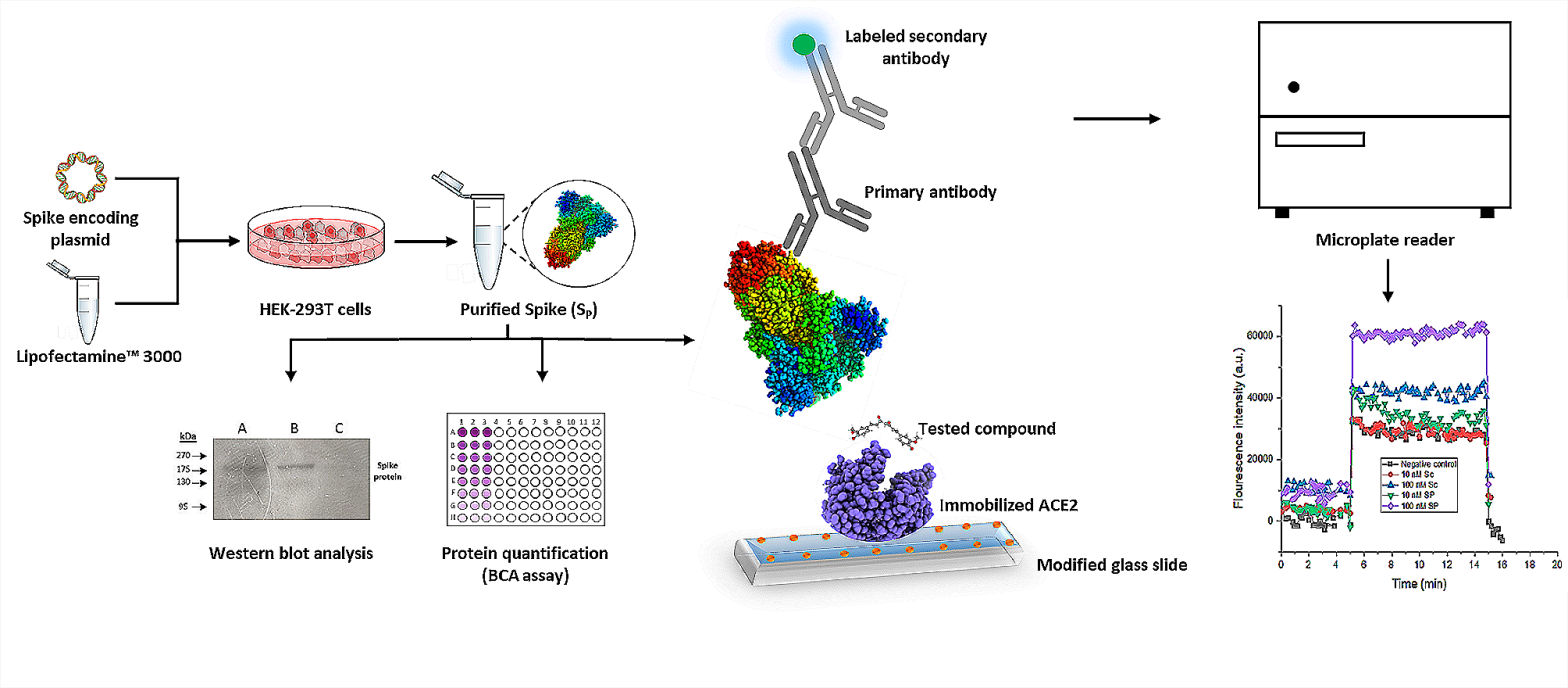

**Supplementary Information:**

The online version contains supplementary material available at 10.1186/s13568-024-01739-8.

## Introduction

Coronavirus,the virus that caused the 2020 pandemic, also known as severe acute respiratory syndrome Coronavirus-2 (SARS-CoV-2) has significantly phased out. However, the efforts to develop and repurpose drugs to be used for the treatment of Coronavirus disease-19 (COVID-19) are still in progress (Lai and Hsueh 2023; Murakami et al. 2023; Saravolatz et al. 2023). To date, the choices for antivirals against SARS-CoV-2 are very limited with only a few FDA-approved drugs. Remdesivir is a broad-spectrum antiviral that inhibits viral replication by terminating ribonucleic acid (RNA) transcription. It was the first drug to be approved in 2020 for the treatment of hospitalized or non-hospitalized patients at high risk for COVID-19 disease progression (Lamb 2020). Following Remdesivir, Tocilizumab (Actemra^®^) and Baricitinib (Olumiant^®^), immunosuppressive drugs used for the treatment of rheumatoid arthritis, were approved in 2022 for the treatment of COVID-19 in hospitalized adults who are receiving systemic corticosteroids and require supplemental oxygen, ventilation, or extracorporeal membrane oxygenation (ECMO) (Assadiasl et al. 2021; Bozorgmanesh et al. 2021).

Spike protein is a surface protein of the SARS-CoV-2 virus that mediates viral adhesion and fusion by interaction with the angiotensin-converting enzyme 2 (ACE2) receptor expressed on the surface of the host cells (Scialo et al. 2020). Spike-ACE2 protein-protein interaction (PPI) is a significant step in virus replication and has been proposed as a target for the discovery of antiviral agents (Jia et al. 2021).

Many research groups have sought to inhibit this PPI using different natural compounds and drugs. Screening methods adopted by several research teams varied, but many used commercial ELISA kits, a sensitive and relatively affordable method (Ahamad et al. 2022; Bojadzic et al. 2021; Tedesco et al. 2022). However, importing such kits to low-income countries is unaffordable and time-consuming.

For this reason, we focused on developing an in-house immunofluorescent assay to screen several inhibitors, with the possibility of repurposing this assay for the screening of ligands, e.g., small molecules, against any other PPI. We sought to exploit a protein-protein interaction detection method that we have developed to detect spike-ACE2 PPI which allowed us to evaluate the binding kinetics of that PPI (Emam et al. 2022). To further increase the affordability of our assay, we opted to produce the spike protein in the lab and use it in the screening assay.

Natural bioactive compounds such as phytochemicals are usually preferred as a treatment option, when applicable, due to their relatively few side effects and sustainable production. Many efforts were made to investigate herbs, herbal extracts, or active compounds for anti-COVID-19 activity (Al-Harrasi et al. 2022; Bizzoca et al. 2022; Chakravarti et al. 2021; Khan et al. 2021). In a previous report, we demonstrated that silymarin, which is a flavonolignan found in *Silybum marianum* (Milk thistle), has an antiviral effect against SARS-CoV-2 and speculated that its mechanism of action may be blocking the viral entry (Loutfy et al. 2022). Gallic acid is a bioactive compound classified as phenolic acid found in many plant families. Gallic acid has shown anti-hepatitis C virus (HCV) and anti-herpes simplex virus-2 (HSV-2) activities in previous literature (Govea-Salas et al. 2016; Jadel Müller Kratz et al. 2008). Quercetin (Q) is a flavonol, which is a polyphenol compound found in plants, with a wide pharmacological activity spectrum ranging from anti-viral and anti-carcinogenic activity to general health improvement and psycho-stimulation ascribed to its potent antioxidant effect (Li et al. 2016; Xu et al. 2019). Quercetin showed an ability to bind to spike protein and ACE2 *in silico* (Hiremath et al. 2021; Pan et al. 2020). Curcumin (Cur) is an active compound derived from *Curcuma longa*. In several clinical trials, it was found to ameliorate inflammation in COVID-19 patients, and it was also proposed to be used as an adjuvant drug in COVID-19 treatment (Rattis, Ramos, and Celes 2021; Vahedian-Azimi et al. 2022).

Here, we have extended the application of our previously published protein-protein interaction detection method (Emam et al. 2022), to screen these selected compounds. The assay was standardized using chitosan nanoparticles (CNPs), a known anti-COVID-19 compound (Loutfy et al. 2022), and the spike-ACE2 interaction model. It was applied to screen the four promising natural compounds: Curcumin (Cur), Gallic acid (GA), Quercetin (Q), and Silymarin (Sil), to elucidate their potential inhibitory activity against spike-ACE2 PPI. We also produced the spike protein whose activity was evaluated and compared with the commercial protein.

## Materials and methods

### Cell lines

Human Embryonic Kidney cells (HEK293T) (ATCC^®^ CRL-3216™) cells and African green monkey kidney (Vero) (ATCC^®^ CCL-81 ™) cells were maintained in Dulbecco’s modified Eagle’s medium (DMEM) (Thermo Fisher Scientific, Gibco™) with 10% FBS (Thermo Fisher Scientific, Gibco™) and 2% Penicillin/Streptomycin (Invitrogen, Inc., Carlsbad, CA).

### Reagents and antibodies

Curcumin (Cur), Gallic acid (GA), Quercetin (Q), Silymarin (Sil), (3-aminopropyl) triethoxysilane (APTES, ≥ 98%), EDC N-(3-dimethylaminopropyl)-N0-ethylcarbodiimide hydrochloride, and N-hydroxy succinimide (NHS) were all purchased from Sigma-Aldrich, USA. MTT (3-[4,5-dimethylthiazol-2-yl]-2,5-diphenyltetrazolium bromide) dye and dimethyl sulfoxide (DMSO) were purchased from Serva Electrophoresis GmbH, Germany. Lipofectamine 3000 reagent (Thermo Fisher Scientific, Invitrogen™) was used for transfection. Spike protein ectodomain encoding plasmid, a generous gift from Dr. Jason S. McLellan (Wrapp et al. 2019), was purified with PureLink™ HiPure Plasmid Maxiprep Kit (K210006, Thermo Fisher Scientific, Invitrogen™). The antibodies used were as follows: Rabbit anti-spike antibody (40,591-T62, Sino biological), spike monoclonal antibody (E-AB-V1008, Elabscience), Goat anti-rabbit antibody (ab6721, Abcam), and Goat anti-human IgG FITC (E-AB-1019, Elabscience). Protein production and quantification were performed by Strep-Tactin^®^XT Spin Column (2-4151-000, iba-lifesciences) and BCA assay kit (23,225, Pierce). Recombinant human ACE2 (PKSH032068, Elabscience) and Recombinant 2019-nCoV spike (PKSR030477, Elabscience) were used in drug screening.

### Plasmid preparation and transfection

The plasmid expressing C-terminal Twin strip tagged spike protein was amplified and purified with a maxiprep kit following the manufacturer’s instructions. HEK293T cells were seeded in a 6-well plate, 6 × 10^5^ cells per well, in DMEM complete media. The following day, the cells were transfected with the plasmid using Lipofectamine 3000 reagent, following the manufacturer protocol. The cells were harvested 72 h post-transfection and prepared for sodium dodecyl sulfate-polyacrylamide gel electrophoresis (SDS-PAGE) analysis.

### Western blot

For the preparation of cell lysates, cells were washed twice with ice-cold PBS, harvested, re-suspended in Laemmli’s buffer, and boiled for 5 min. Samples were separated on 9% SDS‐PAGE and transferred onto polyvinylidene difluoride (PVDF) membranes (Amersham). The membrane was then blocked with 5% non-fat dry milk in TBS and incubated with primary antibody (1:1000) overnight at 4 °C. Following incubation with HRP‐labelled secondary antibody (1:1000) at room temperature, the signal was detected using an ECL kit (Amersham) and visualized using Autoradiography.

### Protein production

For protein production, HEK293T cells were transfected at 70% confluency in 6 × 10 cm plates. Cells were collected 48 h post-transfection and lysed using NP-40 lysis buffer (25 mM Tris-HCl pH 7.4, 150 mM NaCl, 1% NP-40, 5% glycerol) supplemented with protease cocktail inhibitor, and the protein-containing supernatant was collected. Spike protein was purified from the supernatant via affinity purification column and quantified using BCA assay and confirmed with western blot. Produced spike protein was stored at -20 °C in 50% glycerol and thawed upon usage.

### Cell cytotoxicity using MTT assay

MTT assay was performed on Vero cells to evaluate the cytotoxicity of the selected compounds as previously described (Loutfy et al. 2022; Mosmann 1983). Briefly, 10^4^ cells per well were seeded in 96-well plates in DMEM complete media. The following day, cells were treated with different concentrations of the compounds in triplicates. After 48 h, MTT dye was added to the cells (0.5 mg/mL) and incubated for 2 h. The plate was then decanted and 100 µL of DMSO was added per well. Absorbance readings were obtained at 570 nm using a microplate reader (CLARIOstar plus, BMG LabTech, Germany). The cell viability was then expressed as the percentage of the untreated control and CC_50_ (defined as the concentration that reduced cell viability by 50%) values of the tested compounds were calculated by GraphPad Prism 8 software.

### Validation of spike protein binding activity

To validate the binding activity of the produced spike protein (S_P_) versus the commercial spike protein (S_C_) we allowed it to interact with ACE2 using our previously published protein-protein interaction detection method (Emam et al. 2022). Briefly, small glass slides of equal size, 1 cm x 1 cm x 1 mm, were cleaned, coated with APTES, and then activated with EDC-NHS. ACE2 protein was immobilized on the slides, 100 nM on each slide, then they were rinsed with PBS. Both S_P_ and S_C_ were diluted to 2 concentrations 10 nM and 100 nM. Each slide was then incubated with one of the spike proteins followed by the anti-spike antibody (10 nM), then the FITC-labelled secondary antibody (10 nM) with rinsing between each incubation. Two negative controls were included in each run to test for specificity. The negative control was subjected to all the mentioned steps excluding the addition of spike protein.

Fluorescence intensity readings were recorded before and after adding spike protein and after the final wash by transferring slides into 24-well plates and using a microplate reader (CLARIOstar plus, BMG LabTech, Germany) for signal detection.

### Standardization of the in-house immunofluorescent assay for drug screening

Chitosan nanoparticles were used as a drug for the optimization of the assay conditions. CNPs preparation and characterization were previously described (Loutfy et al. 2022). The modified assay was performed following the same procedures as described in the previous section except for an extra incubation step between the immobilized protein (ACE2) and CNPs for 40 min, followed by rinsing with PBS. In the current assay, various concentrations of CNPs (1, 5, 10, 20, 30, and 50 µg/ml) were used (Emam et al. 2021).

Following standardization, we screened the selected compounds: Curcumin (Cur), Gallic acid (GA), Quercetin (Q), and Silymarin (Sil). Taking into consideration the CC_50_ values of each compound, different concentrations ranging from 5 µg/mL to 500 µg/mL were used. The IC_50_ of each compound, which represented 50% inhibition of the substrate (ACE2) binding to its ligand (or to the spike protein), was determined using GraphPad Prism 8 software.

In each run, positive and negative controls were performed. However, the controls were counterintuitive as the negative control included the addition of spike & ACE2 proteins and the antibodies, producing a high signal. While for the positive control, we excluded the addition of spike protein, subsequently, producing no signal.

## Results

### Production of SARS-CoV-2 spike protein

**Fig. 1:**
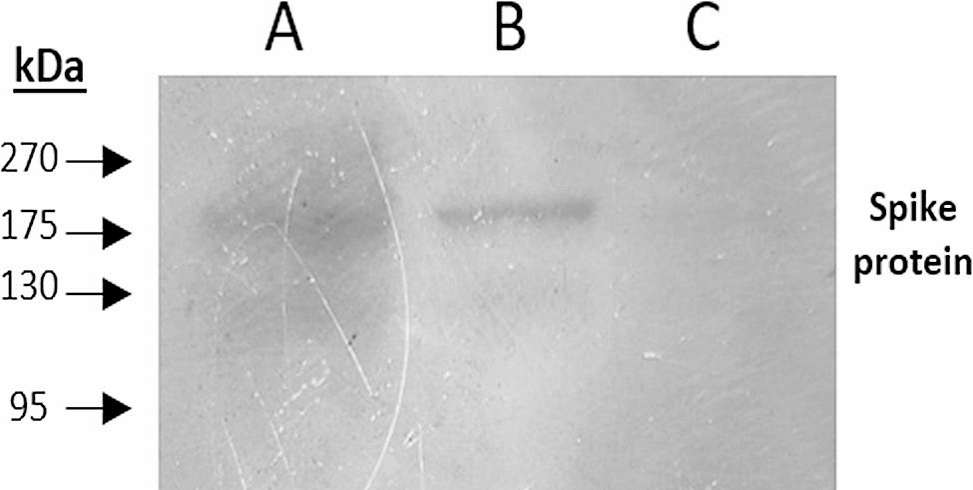
Spike protein expression. Expression of the spike protein was detected using western blot analysis after transfection. Lanes (**A**) and (**B**) represent samples from transfected HEK293T cells, showing bands at 180 kDa corresponding to the spike protein. Lane (**C**) represents the negative control

As an affordable alternative to the commercial spike protein (S_C_), we produced spike protein (S_P_) by transfecting HEK293T cells with spike ectodomain-expressing plasmid. The efficiency of transfection and spike protein expression was determined using western blot analysis. Major bands were observed in lanes A and B (transfected cells) (Fig. 1) at 180 kD corresponding to full-length SARS-CoV-2 spike protein. While no bands were observed in lane C showing the non-transfected negative control. Accordingly, we performed transfection on a larger scale and purified 920 µg of S_P_. (Fig. S1) shows the western blot original autoradiogram.

### Validation of spike protein (S_P_) binding activity to ACE2

**Fig. 2:**
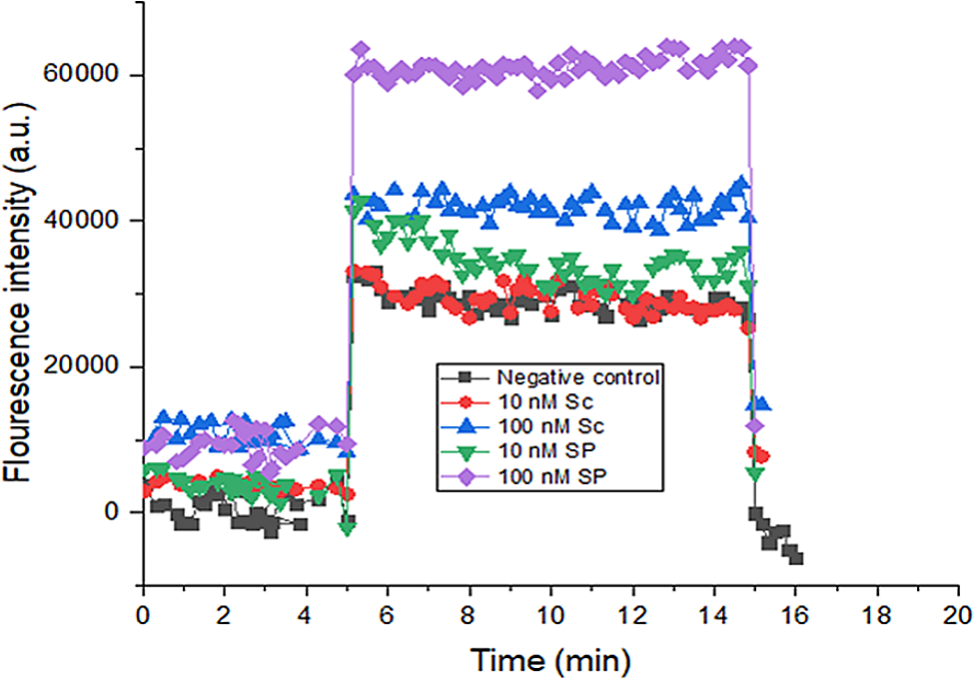
Validation of S_P_ binding activity as compared to S_C_. The fluorescent intensity readings were detected at 2 concentrations for each protein and plotted with Origin software version 2018

In order to compare the binding activity of both the produced S_P_ and the S_C_ to the ACE2 protein, we tested each at 2 concentrations: 10 and 100 nM via immunofluorescent assay. Both S_P_ and S_C_ produced similar signals when detected at each of the 2 concentrations (Fig. 2), proving that S_P_ binding activity remained intact after the purification process.

### Determination of the cytotoxicity of the four natural compounds using MTT

CNPs, Cur, GA, Q, and Sil were tested and their CC_50_ values were detected at 175, 13, 18, 73, and 65 µg/mL, respectively (Table 1).

**Table 1:** CC_50_ values of the screened compounds on Vero cells

No.	Compound	CC_50_ (µg/mL)
1	CNPs	175
2	Curcumin	13
3	Gallic acid	18
4	Quercetin	73
5	Silymarin	65

### Standardization of in-house immunofluorescent assay for the determination of the inhibitory effect of selected compounds

**Fig. 3:**
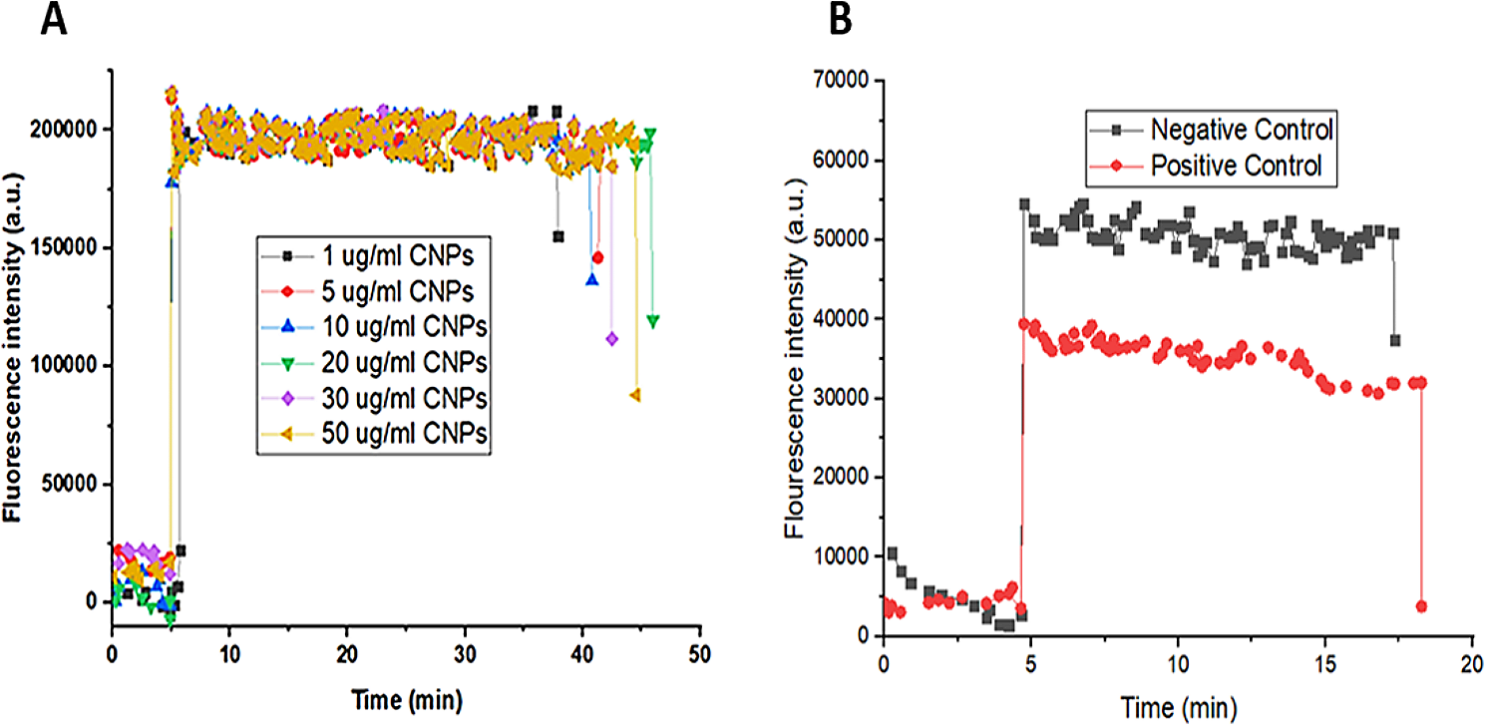
Standardization of the in-house immunofluorescent assay. (**A**) The effect of different CNPs concentrations on the signal intensity. The reduction in the signal corresponds to the increase in CNPs concentration, and (**B**) The negative and positive controls, the positive control (containing ACE2 only) shows no signal while the negative control (containing spike and ACE2) shows a high signal

As the ability of CNPs to inhibit spike-ACE2 interaction was previously identified (Loutfy et al. 2022), they were used in the standardization of the new immunofluorescent assay for drug screening. Different concentrations of CNPs were screened using the modified protocol of our protein-protein interaction detection assay (Emam et al. 2022). The signal was decreased after the rinsing of the labeled-secondary antibody step, in which the variation in fluorescence signals was linearly dependent on the concentration of the CNPs (Fig. 3A) proving the specificity of the observed binding.

This was further proved by the controls. The negative control (containing spike and ACE2) curve shows a high fluorescence intensity that corresponds to the binding between the ACE2 and the spike protein. In contrast, the positive control (containing ACE2 only) curve shows decreased fluorescence intensity which indicates the absence of spike-ACE2 interaction (Fig. 3B). Therefore, inhibition of spike-ACE2 interaction resembles the positive control here.

### Screening of four bioactive natural compounds using the standardized immunofluorescent assay

**Fig. 4:**
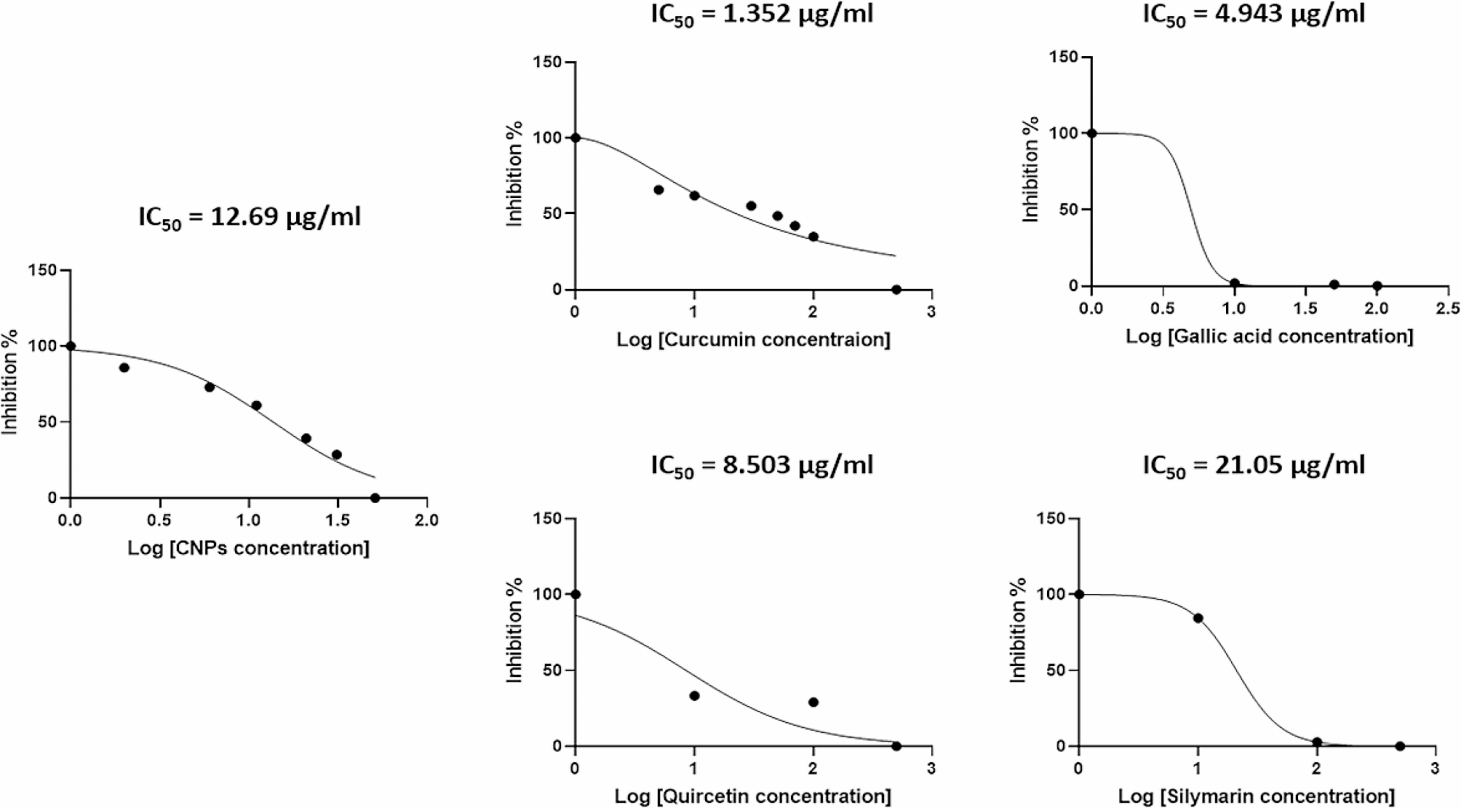
Inhibitory effect of screened compounds. The drug-response curve was calculated for each drug with GraphPad Prism 8 software. IC_50_ values of CNPs, Cur, GA, Q, and Sil were detected at 12.69, 1.352, 4.943, 8.503, and 21.05 µg/mL

Results showed that the signal representing spike-ACE2 PPI was markedly hindered after treatment with any of the tested compounds. Hence the decrement of the signal corresponded to the increase in the compounds’ concentrations, indicating their ability to disrupt spike-ACE2 PPI. The compounds’ inhibitory effect was represented as IC_50_ values (Fig. 4). CNPs, Cur, GA, Q, and Sil IC_50_ values were determined at 12.69, 1.352, 4.943, 8.503, and 21.05 µg/mL, respectively.

## Discussion

The development of new laboratory methods that are simple enough to facilitate the drug discovery process is important and of high value, especially during pandemics when multiple research groups are working simultaneously and require affordable methods.

This study aimed to explore the applicability of our immunofluorescent protein-protein interaction (PPI) detection method for drug screening (Emam et al. 2022). Focusing on the well-known SARS-CoV-2 Spike protein and Angiotensin Converting Enzyme 2 (ACE2) interaction, we standardized the assay using chitosan nanoparticles (CNPs) as a known inhibitor (Loutfy et al. 2022). The successful screening of CNPs against Spike-ACE2 interaction allowed us to calculate the IC_50_ value, representing 50% inhibition of tested protein-protein interaction. We also sought to reduce screening costs by producing the spike protein (S_P_) in HEK293T cells, ensuring its binding activity to ACE2 was comparable to the commercial spike protein (S_C_).

It has been reported that some natural compounds are promising candidates for curing many diseases. Their significant pharmacological effects and mode of action as well as their low toxicity allowed them to be thought of as treatments for COVID-19 (Al-Harrasi et al. 2022; Bizzoca et al. 2022; Chakravarti et al. 2021; Khan et al. 2021). Therefore, After the standardization of the screening assay and spike protein production, we selected four natural compounds, Curcumin (Cur), Gallic acid (GA), Quercetin (Q), and Silymarin (Sil), as candidates to be screened using our assay. We assessed their cytotoxicity using MTT assay on Vero cells to obtain CC_50_ values, representing 50% reduction of the cell viability, before screening for their inhibitory activity.

Our results were in agreement with other reported results. Low and colleagues reported that concentrations of Silymarin up to 200 µg/mL did not show significant toxicity on Vero cells (Low et al. 2021). Furthermore, Ortiz-Andrade et al. evaluated the cytotoxic effect of Quercetin and reported its safety on Vero cells, and that only high concentrations ranging from 250 to 750 µg/mL decreased the cellular viability (Ortiz-Andrade et al. 2020). Additionally, Marin-Palma reported that CC_50_ of Curcumin on Vero E6 cells was obtained at 16.5 µg/mL (Marín-Palma et al. 2021). Moreover, Abdullah and colleagues determined the antiproliferative activity of Gallic acid to be 10.00 ± 1.06 µg/mL on HeLa cells (Abdullah et al. 2023).

After cytotoxicity evaluation of the selected natural compounds, they were screened using the standardized protocol for drug screening using our immunofluorescent assay. All the screened compounds were able to effectively disrupt the Spike-ACE2 interaction, with Curcumin demonstrating the strongest inhibitory effect, having the lowest IC_50_ value (1.352 µg/mL) which is even lower than that of our positive control, CNPs (12.69 µg/mL).

This is supported by previously reported data about the anti-viral effect of Curcumin against viruses, especially SARS-CoV-2. Curcumin demonstrated antiviral activity against influenza A virus, SARS-CoV, human immunodeficiency virus (HIV), enterovirus 71 (EV71), herpes simplex virus (HSV), hepatitis C virus (HCV), human papillomavirus (HPV), Zika virus, and Chikungunya virus; Multiple mechanisms contributed to its antiviral activity (Babaei, Nassiri-Asl, and Hosseinzadeh 2020; Soni et al. 2020). Furthermore, in-silico studies predicted the binding of curcumin with receptors that mediate viral entry of SARS-CoV-2 into host cells (Soni et al. 2020).

We can see that, this approach provides an easy and rapid screening tool for anti-SARS-CoV-2 candidates. However, the main limitation of our assay lies in its inapplicability to High Throughput Screening (HTS). This limitation arises from its reliance on glass slides that need to be transferred to a plate for microplate reader analysis, unlike ELISA assays that can be directly performed in a 96-well plate. Despite this limitation, our study contributes a valuable tool using cost-effective and efficient screening method for drugs under investigation.

In summary, our study successfully demonstrated the feasibility of utilizing our previously established immunofluorescent assay for drug screening against the Spike-ACE2 interaction. We produced a cost-effective in-house spike protein, which was highly comparable to the commercial one. The screening of natural compounds, particularly Curcumin, showed promising inhibitory effects with acceptable safety profiles. While the assay has limitations concerning HTS, its cost-effectiveness, and reproducibility make it a valuable tool for targeted drug screening in the context of specific protein-protein interaction. Further research is needed to confirm the antiviral effects in vitro and in vivo of the screened compounds, determining their potential as additional drugs for COVID-19 treatment.

## Electronic supplementary material

Below is the link to the electronic supplementary material.

Supplementary Material 1
