# Supplementary material for: Establishment of in-house assay for screening of anti-SARS-CoV-2 protein inhibitors
Source: AMB Express. 2024 Sep 16;14:104. doi: 10.1186/s13568-024-01739-8 (PMC11405717; doi:10.1186/s13568-024-01739-8)
Supplement: Supplementary file 1 — Supplementary Material 1 [file 13568_2024_1739_MOESM1_ESM.docx]

Establishment of in-house IF assay for screening of anti-SARS-CoV-2 PPI inhibitors

*Merna H. Emam^1^, Mohamed I. Mahmoud^1,2^, Nadia El-Guendy^3^, Samah A. Loutfy^1,4^*^*^

*^1^Nanotechnology Research Center (NTRC), the British University in Egypt, El-Shorouk City, Suez Desert Road, Cairo 11837 -P.O. Box 43, Egypt.*

*^2^School of Biotechnology, Badr University in Cairo, Badr City, Cairo 11829, Egypt.*

*^3^Biochemistry unit, Cancer Biology Department, National Cancer Institute (NCI), Cairo University, Fom El-Khalig 11796, Cairo, Egypt.*

*^4^Virology and Immunology Unit, Cancer Biology Department, National Cancer Institute (NCI), Cairo University, Fom El-Khalig 11796, Cairo, Egypt
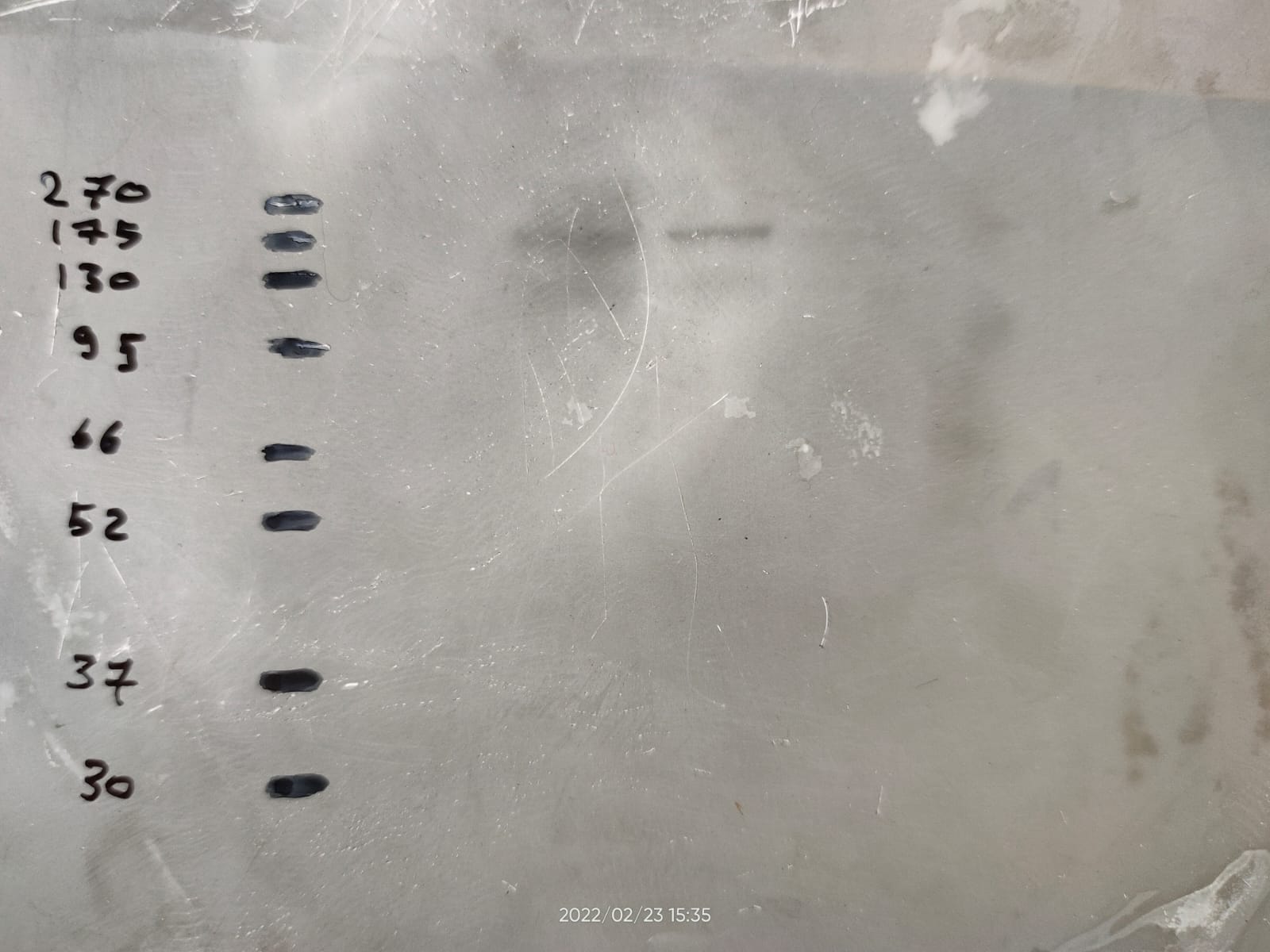
*

Fig. S1. The western blot original autoradiogram (raw data).
